# Differences in the relative importance of predictors of short- and long-term mortality among critically ill patients with cancer

**DOI:** 10.62675/2965-2774.20240149-en

**Published:** 2024-11-11

**Authors:** Carla Marchini Dias da Silva, Bárbara Beltrame Bettim, Bruno Adler Maccagnan Pinheiro Besen, Antônio Paulo Nassar

**Affiliations:** 1 A.C. Camargo Cancer Center Intensive Care Unit São Paulo SP Brazil Intensive Care Unit, A.C. Camargo Cancer Center - São Paulo (SP), Brazil.; 2 A. C. Camargo Cancer Center International Research Center São Paulo SP Brazil International Research Center, A. C. Camargo Cancer Center - São Paulo (SP), Brazil.

**Keywords:** Critical illness, Neoplasms, Critical care outcomes, Mortality, Prognosis, Intensive care units

## Abstract

**Objective:**

To identify the relative importance of several clinical variables present at intensive care unit admission on the short- and long-term mortality of critically ill patients with cancer after unplanned intensive care unit admission.

**Methods:**

This was a retrospective cohort study of patients with cancer with unplanned intensive care unit admission from January 2017 to December 2018. We developed models to analyze the relative importance of well-known predictors of mortality in patients with cancer admitted to the intensive care unit compared with mortality at 28, 90, and 360 days after intensive care unit admission, both in the full cohort and stratified by the type of cancer when the patient was admitted to the intensive care unit.

**Results:**

Among 3,592 patients, 3,136 (87.3%) had solid tumors, and metastatic disease was observed in 60.8% of those patients. A total of 1,196 (33.3%), 1,738 (48.4%), and 2,435 patients (67.8%) died at 28, 90, and 360 days, respectively. An impaired functional status was the greatest contribution to mortality in the short term for all patients and in the short and long term for the subgroups of patients with solid tumors. For patients with hematologic malignancies, the use of mechanical ventilation was the most important variable associated with mortality in all study periods. The SOFA score at admission was important for mortality prediction only for patients with solid metastatic tumors and hematological malignancies. The use of vasopressors and renal replacement therapy had a small importance in predicting mortality at every time point analyzed after the SOFA score was accounted for.

**Conclusion:**

Healthcare providers must consider performance status, the use of mechanical ventilation, and the severity of illness when discussing prognosis, preferences for care, and end-of-life care planning with patients or their families during intensive care unit stays.

## INTRODUCTION

The cancer death rate has been declining worldwide, with a 33% overall reduction since 1991.^(1)^ This progress increasingly reflects improvements in early detection as well as advances in treatment protocols, including the development of targeted therapies.^(2)^ However, these changes have also been accompanied by an increased number of patients susceptible to life-threatening complications. Consequently, a growing number of cancer patients are at risk of admission to the intensive care unit (ICU).^(3,4)^ The risk of ICU admission due to medical complications of cancer treatment is 15.0%,^(5)^ and up to 22.5% of patients with hematologic malignancies need ICU care during the first year after diagnosis.^(6)^

Recent major changes in the landscape of oncology have raised questions about current indications for ICU admission and the related prognosis in critically ill patients with cancer. Although outcomes in critically ill patients with cancer have improved in the last decade, ICU care is associated with persistent morbidity after discharge,^(7)^ and patients with cancer perceived by intensivists as potentially inappropriate for ICU admission have a one-year mortality of 97.6%.^(8)^

The decision about whether to admit a patient to the ICU can be a difficult clinical and ethical challenge. Several studies have assessed the influence of risk factors associated with short-term outcomes (ICU, hospital, and 30-day mortality);^(9,10)^ however, few have evaluated their impact on long-term mortality in patients with cancer after ICU admission. A better understanding of the factors that potentially influence patients’ outcomes can help healthcare professionals improve their shared decision-making. To help bridge these gaps, our study aimed to identify the relative importance of several clinical variables present at ICU admission on the short- and long-term mortality of critically ill patients with cancer after unplanned ICU admission.

## METHODS

### Study design and setting

This was a secondary analysis of a retrospective cohort study on prospectively collected data conducted from January 2017 to December 2018 in a dedicated cancer center in Brazil.^(11)^ The Ethics Committee for Scientific Research of the A. C. Camargo Cancer Center approved the study and waived the need for informed consent since all the data were fully anonymized before researchers obtained access (CAAE: 05694819.3.0000.5432).

Our center is a 447-bed tertiary care referral center for cancer patients in São Paulo, Brazil, and it provides care for approximately 1,850 medical and surgical patients per month. The Critical Care Department consists of five medical-surgical ICUs and has a total of 50 ICU beds. All ICUs have the same profile and can admit patients from the emergency department, operating room, wards, or patients transferred from another hospital. There is no ICU triage policy in the hospital. After the patient is admitted, the ICU team is ultimately responsible for patient care. Decisions regarding life-sustaining treatment and ICU discharge are made by the intensivist in charge. Decisions on withholding or withdrawing life-sustaining therapies are made after discussions among intensivists, oncologists/hematologists, and patients or the patient's next of kin.

We report this study in accordance with the Transparent Reporting of a Multivariable Prediction Model for Individual Prognosis or Diagnosis (TRIPOD) statement.^(12)^

### Study population and data collection

We included all adult patients (age ≥ 18 years) with unplanned ICU admissions for this analysis. We excluded patients without cancer and those readmitted to the ICU during the same hospital stay.

We retrieved patient data from local databases and electronic medical records. The following demographic and clinical data were collected: type of cancer (solid local/regional, solid metastatic, or hematologic); site of solid cancer or type of hematologic malignancy; Eastern Cooperative Oncology Group Performance Status (ECOG PS)^(13)^ before hospital admission (registered at the last outpatient clinic visit); Charlson comorbidity index (CCI),^(14)^ Sequential Organ Failure Assessment (SOFA) score^(15)^ and Simplified Acute Physiology Score III (SAPS III)^(16)^ at admission, and reason for admission to the ICU.

Additionally, we collected data on the use of organ support (vasopressor therapy, invasive mechanical ventilation [MV], renal replacement therapy [RRT]), ICU and hospital length of stay (LOS), and mortality at 28 days, 90 days, and 360 days.

### Predictors and outcomes

We included well-known predictors of mortality in patients with cancer admitted to the ICU: type of cancer (solid local/regional, solid metastatic, or hematologic), age, comorbidity burden assessed through the CCI (without points related to cancer), SOFA score, use of vasopressors, need for MV and RRT, and ECOG performance status.^(3,17–19)^

The relative importance of predictors was evaluated against mortality at 28, 90, and 360 days after ICU admission both in the full cohort and stratified by the type of cancer at ICU admission.

### Statistical analysis

#### Model development

The predictions of mortality models were developed in the study population cohort. The incidence of mortality was regressed on age and the prespecified predictor variables. All predictor numeric variables (age, CCI and SOFA) were modeled as continuous variables.^(20)^ We assessed the relative variable importance with each variable's Wald chi-square test relative to the overall chi-square test in the multivariable model. Model coefficients (and odds ratios) were derived, but as they were not the primary presentation of interest, we did not perform shrinkage or variable selection procedures. The same modeling strategy was applied across the four cohorts (full cohort, solid locoregional cancers, solid metastatic cancers, and hematological cancers) and for each mortality time point (28 days, 90 days and 360 days). We evaluated the apparent validation for these models with the C statistic (area under the receiving operator characteristics curve)^(21)^ and calibration plots.^(22)^ We also performed internal validation with optimism-corrected estimates of discrimination after 200 bootstrap resamples.^(20)^

Among the selected variables used in the models, only the ECOG data (11%) were missing. We assumed data to be missing at random and applied a multiple imputation technique using chained equations (MICEs).^(23)^

All analyses were performed using the Statistical Package for the Social Sciences (SPSS) version 20 and R version 4.3.0 (R Foundation for Statistical Computing).

## RESULTS

### Characteristics of the study population

**Figure 1:**
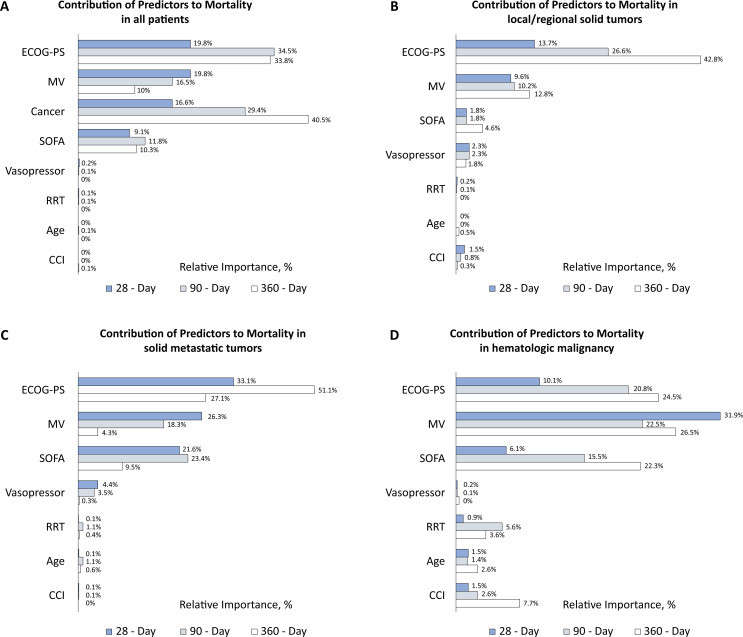
Relative predictor importance for each mortality timepoint stratified by type of cancer

Between January 2017 and December 2018, 8,064 patients were admitted to the ICUs. Of these, 3,592 patients were eligible for the analyses (Figure 1S - Supplementary Material). The mean age was 65 (± 15) years, 1,747 (48.6%) were women, and 3,136 (87.3%) had solid tumors. The colorectal (14.8%), lung (12.1%), breast (11.1%), and head and neck (7.8%) regions were the most common sites of solid tumors. Metastatic disease was observed in 60.8% of those patients. Non-Hodgkin lymphoma (33.9%), multiple myeloma (18%), and acute leukemia (17.7%) were the most common hematologic malignancies.

There were 3,205 patients (89.2%) admitted because of medical complications and 387 (10.8%) following urgent surgeries. The main reasons for medical admissions were sepsis and septic shock (30.5%), acute respiratory failure (ARF) (19.1%), and neurological disorders (6.5%). The mean SOFA score was 3.0 (± 3) points, the median CCI score was 7.0 (5 - 8) and the the mean SAPS 3 score was 64 (± 15).

The median lengths of ICU and hospital stays were 5 (2 - 10) and 12 (6 - 24) days, respectively. A total of 1,196 (33.3%), 1,738 (48.4%), and 2,435 patients (67.8%) died at 28, 90, and 360 days, respectively (Table 1).

**Table 1:** Characteristics of patients with unplanned intensive care unit admission

Variable		
Female,		1,747 (48.6)
Age (years)		62 ± 15
CCI		7 [5 - 8]
ECOG*		3,297 (97.6)
	0	727 (22.1)
	1	1,365 (41.4)
	2	694 (21.0)
	3	440 (13.3)
	4	71 (2.2)
SOFA		3 ± 3
SAPS 3		64 ± 15
Solid tumor		3,136 (87.3)
Solid, metastatic		1,909 (60.8)
Hematologic malignancy		456 (12.7)
Admission type		
	Medical	3,205 (89.2)
	Urgent surgery	387 (10.8)
Reason for admission		
	Sepsis and septic shock	979 (30.54)
	Acute respiratory failure	613 (19.13)
	Altered mental status	209 (6.52)
	Arrhythmia	147 (4.59)
Renal replacement therapy		92 (2.6)
Mechanical ventilation		533 (14.8)
Vasopressors		1,082 (30.1)
ICU length of stay (days)		5 [2 - 10]
Hospital length of stay (days)		10 [5 - 19]
28-day mortality		1,196 (33.3)
90-day mortality		1,738 (48.4)
360-day mortality		2,435 (67.8)

CCI - Charlson Comorbidity Index; ECOG - Eastern Cooperative Oncology Group; SOFA - Sequential Organ Failure Assessment; SAPS - Simplified Acute Physiological Score; ICU - intensive care unit.

* Data were missing for 395 (11%) patients. The results are expressed as n (%), means ± standard deviations, or medians [interquartile ranges].

### Relative importance of different predictors of mortality in patients admitted to an intensive care unit

#### All patients

Performance status was the most important variable associated with mortality at 90 days in all patients and had the highest relative importance, along with MV, at 28 days. Its relative importance increased from 19.8% at 28 days to 34.5% at 90 days, and subsequently decreased to 33.8% at 360 days. On the other hand, the type of cancer was the most important variable associated with mortality at 360 days. The relative importance of cancer increased from 16.6% at 28 days to 29.4% and 40.5% at 90 and 360 days, respectively. The relative importance of MV decreased from 19.8% at 28 days to 16.5% and 10% at 90 and 360 days, respectively. The relative importance of the SOFA score at admission for mortality prediction at 28 days was 9.1%, which subsequently increased to 11.8% at 90 days and then decreased to 10.3% at 360 days. The use of vasopressors, RRT, age, and comorbidities other than cancer had few relative importance in predicting mortality (Figure 1A).

The relative importance of each predictor is estimated as the ratio of the Wald chi-square test for the predictor to the overall Wald chi-square test.

ECOG-PS - Eastern Cooperative Oncology Group performance status; MV - mechanical ventilation; SOFA - Sequential Organ Failure Assessment; RRT - renal replacement therapy; CCI - Charlson comorbidity index.

#### Patients with local/regional solid tumors

Performance status was the most important variable associated with mortality at 28, 90, and 360 days in patients with local/regional solid tumors. Its relative importance increased from 13.7% at 28 days to 26.6% and 42.8% at 90 days and 360 days, respectively. Mechanical ventilation was the second most important variable for mortality prediction, with values of 9.6%, 10.2%, and 12.8% at 28, 90, and 360 days, respectively. The relative importance of the SOFA score at admission for mortality prediction at 28 and 90 days was 1.8%, which increased to 4.6% at 360 days. The use of vasopressors had a relative importance of 2.3%, 2.3%, and 1.8% for mortality prediction at 28, 90, and 360 days, respectively. Comorbidities other than cancer had a relative importance of 1.5% for mortality prediction at 28 days, with negligible importance at 90 and 360 days. Renal replacement therapy and age had few relative importance in predicting mortality during all the study periods (Figure 1B).

#### Patients with metastatic solid tumors

Performance status was the most important variable associated with mortality at 28, 90, and 360 days in patients with metastatic solid tumors. Its relative importance increased from 33.1% at 28 days to 50.1% at 90 days and subsequently decreased to 27.1% at 360 days. Mechanical ventilation was the second most important variable for mortality prediction at 28 days, with a decrease in importance from 26.3% to 18.3% and to 4.3% at 28, 90, and 360 days, respectively. The SOFA score was the second most important variable for mortality prediction at 90 and 360 days. Its relative importance increased from 21.6% at 28 days to 23.4% at 90 days and subsequently decreased to 9.5% at 360 days. The use of vasopressors had a relative importance of 4.4% and 3.5% at 28 and 90 days, respectively. Renal replacement therapy, age, and comorbidities other than cancer had a relative importance of less than 1.5% in mortality prediction during all study periods (Figure 1C).

#### Patients with hematologic malignancies

Mechanical ventilation was the most important variable associated with mortality in all the study periods. Its relative importance decreased from 31.9% at 28 days to 22.5% at 90 days and subsequently increased to 26.5% at 360 days. The performance status was the second most important variable associated with mortality, and its relative importance increased from 10.1% at 28 days to 20.8% and 24.5% at 90 and 360 days, respectively. The relative importance of the SOFA score at admission for mortality prediction increased from 6.1% at 28 days to 15.5% and 22.3% at 90 days and 360 days, respectively. Comorbidities other than cancer had relative importance of 1.5%, 2.6%, and 7.7% for mortality prediction at 28, 90, and 360 days, respectively. The relative importance of RRT increased from 0.9% at 28 days to 5.6% at 90 days and then decreased to 3.6% at 360 days. The use of vasopressors and age had a relative importance of less than 1% for mortality prediction at all study periods (Figure 1D).

All the models presented good discrimination (Table 1S - Supplementary Material). The estimated odds ratios and 95% confidence intervals are presented in the supplemental file (Tables 2S to 5S - Supplementary Material).

## DISCUSSION

The results of this study show that an impaired functional status provided the greatest contribution to mortality in the short term for all patients and in the short and long term for the subgroups of patients with solid tumors. For patients with hematologic malignancies, the use of MV was the most important variable associated with mortality in all study periods. The SOFA score at admission was relatively important for mortality prediction only for patients with solid metastatic tumors and hematological malignancies. The use of vasopressors and RRT had a small relative importance in predicting mortality at every time point analyzed after the SOFA score was accounted for.

Our analysis confirmed the role of an impaired performance status in poor outcomes in patients with cancer, contributing to the greatest risk for mortality, especially in patients with solid tumors. Pre-existing impaired functionality is associated with worse outcomes during and after ICU admission,^(3,7,19)^ and recent research has indicated that recovering from a critical illness deteriorates the baseline functional capacity in patients with cancer,^(7)^ which may impact candidacy for ongoing treatments,^(24,25)^ and may be associated with lower survival up to 1 year after hospital discharge.^(24,26)^

Although recent studies have shown that mortality in patients with cancer who use mechanical ventilation during their ICU stay have improved over time,^(3,10)^ our findings indicate that MV is still an important prognostic factor. In fact, in patients with hematologic malignancies, the use of mechanical ventilation represented the variable associated with the highest relative importance to mortality at every point analyzed. Acute respiratory failure is still the major cause of ICU admission for these patients,^(27)^ and the need for MV continues to be a determinant of worse outcomes,^(27,28)^ despite improvements in the prognosis of critically ill patients with hematologic cancer over time.^(3,10,29)^ Its high mortality has led physicians to consider noninvasive ventilation (NIV) to improve the prognosis of this population. However, the best initial strategy for the ventilatory management of these patients still raises doubts and uncertainties.^(26,29,30,31)^ Although previous studies have demonstrated that NIV can benefit hematological malignancy patients with ARF,^(30,31)^ it may fail in approximately 50% of patients,^(28,30)^ and those who fail NIV have a similar or even worse prognosis than patients who initially received MV.^(28,30,31)^ Early ICU admission, etiological investigation of hypoxemic respiratory failure, and identification of ideal NIV candidates among oncohematological patients can help maximize the effectiveness of this tool in cases of respiratory dysfunction within this population.^(25,27)^

Our study suggests that age and the comorbidity burden beyond those associated with cancer had negligible effects on mortality risk at every time point analyzed. These findings are not unexpected. Compared with undifferentiated cohorts of patients with cancer admitted to the ICU, very old patients with cancer admitted to the ICU have similar short- and long-term mortality rates.^(32,33)^ Although the comorbidity burden is a significant predictor of long-term mortality in critically ill patients,^(34)^ much of the comorbidity impact in this study was due to cancer *per se*.^(32)^ Curiously, the comorbidity burden was of some relative importance for 360-day outcomes for the hematological cancer patients in our cohort.

The SOFA score at admission was relatively important for mortality only for patients with solid metastatic tumors and hematological malignancies, and the subgroup of patients in which mechanical ventilation was used had a relatively high proportional weight for mortality prediction, although the relative importance of the SOFA score increased for hematological malignancies in the long term but decreased for solid metastatic tumors. In the general population, this association between the SOFA score and worse outcomes is stronger in the short term and wanes through time, whereas the relative importance of the comorbidity burden increases in the long term.^(35)^ In our study, although the non-oncological comorbidity burden was not associated with long-term survival, cancer characteristics and poor performance status were associated.

Studies have shown that the use of vasopressors and the need for RRT during an ICU stay are independently associated with increased mortality, and patients with cancer who require dialysis have in-hospital mortality between 51 and 90%.^(36–38)^ Interestingly, the use of vasopressors and RRT contributed relatively little to mortality, even 28-day mortality. This likely occurred because our model accounted for the SOFA score. Our results suggest that outcomes are affected not only by single factors but by a complex interplay among the different types of cancer, the level of organ support, a proxy for the severity of acute illness, and performance status. This has been observed in other specific populations, such as very elderly patients with pneumonia undergoing invasive MV or NIV^(39)^ or critically ill trauma patients, where the SOFA score is a highly reliable composite predictor of outcome.^(40)^ Therefore, the need for vasopressors or RRT should not be an isolated factor to deny an increase in ICU-level care or to initiate comfort care and end-of-life care discussions in the ICU, especially for patients with cancer and with good functional capacity.

Our study has important strengths. First, we specifically assessed patients with cancer, in whom clinicians may have doubts about the appropriateness of ICU admission. High-quality research concerning the short- and long-term mortality of patients with cancer with unplanned ICU admission is essential to manage the outcome expectations of healthcare providers, patients, and their relatives. Second, we analyzed mortality and prognostic factors by stratifying patients according to different types of cancer (solid local/regional, solid metastatic, or hematologic). Our finding of differences in the importance of prognostic factors between the malignancy groups indicates that a patient's prognosis should be predicted without merging these groups under a cancer umbrella. Moreover, the ability of the combined prognostic factors to predict the effects of several clinical variables present at ICU admission on the short- and long-term mortality of critically ill patients with cancer is notable and worth validating in future studies.

Our study also has several limitations. First, as it is a single-center study conducted in a dedicated cancer center, its results may not be generalizable. Second, we were unable to include data on frailty, which is independently associated with outcomes and resource use in critically ill patients and could have impacted the results.^(41)^ Third, although we excluded patients with a defined directive on forgoing life-sustaining therapy before ICU admission, we did not consider decisions on forgoing life-sustaining therapies throughout the ICU stay, which could have impacted short- and possibly long-term mortality.^(42)^ Fourth, we did not collect data on the time between ICU admission, the development of organ dysfunctions, and the start of organ-support therapies. However, at our hospital, ICU beds are immediately available to patients in need of organ support. Fifth, we did not collect data on socioeconomic status, which is associated with outcomes and could have impacted the results. Lower individual-level and area-level socioeconomic status is associated with suboptimal cancer care and reduced survival^(43)^ since patients with cancer from socioeconomically deprived areas have limited access to screening and treatment services and tend to have more advanced disease at presentation.^(44)^ Sixth, we did not collect data on quality of life. The significant burden of physical, cognitive, and mental health impairments after ICU discharge, coupled with increased vulnerability to adverse outcomes such as mortality and rehospitalization, cannot be overlooked.^(45)^ Finally, we did not account for other individual cancer characteristics that may be used in individual decision-making, which should likely be considered in addition to our observed results.

## CONCLUSION

Our study revealed that clinical variables present at intensive care unit admission have a complex interaction and have different importance in the mortality of patients with cancer after unplanned intensive care unit admission according to different types of cancer and different mortality landmarks. Healthcare providers must consider the performance status, use of mechanical ventilation, and severity of illness when discussing prognosis, preferences for care, and end-of-life care planning with patients or their families during an intensive care unit stay. However, further studies are still needed to characterize predictors of other long-term outcomes in critically ill patients with cancer.

## Data Availability

The datasets used and analyzed in our study are available from the corresponding author upon reasonable request.
