# Supplementary material for: Differences in the relative importance of predictors of short- and long-term mortality among critically ill patients with cancer
Source: Crit Care Sci. 2024 Nov 11;36:e20240149en. doi: 10.62675/2965-2774.20240149-en (PMC11634285; doi:10.62675/2965-2774.20240149-en)
Supplement: Supplementary file 1 [file 2965-2774-ccsci-36-e20240149en-Suppl01.pdf]

Critical Care Science
https://doi.org/10.62675/2965-2774.20240149-en

SUPPLEMENTARY MATERIAL

Differences in the relative importance of predictors of short- and long-term mortality among critically ill patients with cancer

Carla Marchini Dias da Silva[1], Bárbara Beltrame Bettim[2], Bruno Adler Maccagnan Pinheiro Besen[1], Antônio Paulo Nassar Junior[1]

SUMMARY

Figure 1S - Study flowchart 2
Table 1S - Bootstrap internal validation with optimism-correct areas under the receiving-operator characteristic curve 2
Table 2S - Estimated odds ratios and 95% confidence intervals of individual predictors for all patients 3
Table 3S - Estimated odds ratios and 95% confidence intervals of individual predictors for all locoregional solid tumors 3
Table 4S - Estimated odds ratios and 95% confidence intervals of individual predictors for metastatic solid tumors 4
Table 5S - Estimated odds ratios and 95% confidence intervals of individual predictors for hematological malignancy 4

This is an open access article under the CC BY license (https://creativecommons.org/licenses/by/4.0/).

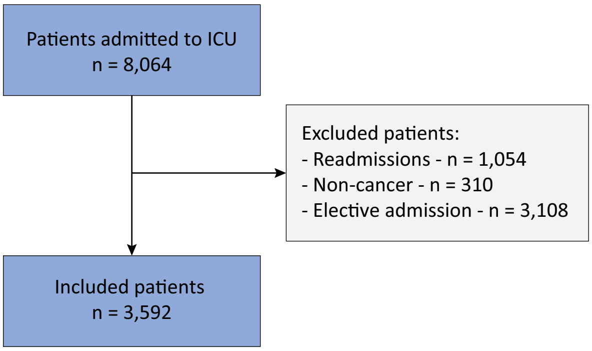

Figure 1S - Study participants.
ICU - intensive care unit.

Table 1S - Bootstrap internal validation with optimism-correct areas under the receiving-operator characteristic curve

	AUROC (95%CI)
All patients	
28-day mortality	0.801 (0.800 - 0.802)
90-day mortality	0.797 (0.796 - 0.798)
360-day mortality	0.795 (0.794 - 0.796)
Locorregional solid tumors	
28-day mortality	0.805 (0.803 - 0.808)
90-day mortality	0.786 (0.784 - 0.788)
360-day mortality	0.768 (0.766 - 0.770)
Metastatic solid tumors	
28-day mortality	0.756 (0.755 - 0.758)
90-day mortality	0.751 (0.749 - 0.752)
360-day mortality	0.741 (0.739 - 0.743)
Hematologic malignancy	
28-day mortality	0.851 (0.848 - 0.854)
90-day mortality	0.828 (0.825 - 0.831)
360-day mortality	0.763 (0.759 - 0.766)

AUROC - area under the receiving operator characteristics curve; 95%CI - 95% confidence interval.

Table 2S - Estimated odds ratios and 95% confidence intervals of individual predictors for all patients

Variable	28-day		90-day		360-day	
	OR	CI95%	OR	CI95%	OR	CI95%
Solid tumor, locoregional	Ref		Ref		Ref	
Solid tumor, metastatic	3.59	2.91 - 4.46	4.06	3.36 - 4.91	4.78	4.00 - 5.72
Hematological malignancy	4.06	3.36 - 4.91	1.65	1.25 - 3.17	1.57	1.22 - 2.03
ECOG-OS						
0 - 1	Ref		Ref		Ref	
2	2.48	2.01 - 3.05	2.91	2.39 - 3.55	2.65	2.15 - 3.29
3 - 4	4.01	3.19 - 5.05	4.44	3.53 - 5.63	3.89	3.01 - 5.07
SOFA	1.17	1.12 - 1.21	1.17	1.13 - 1.22	1.17	1.12 - 1.22
MV	6.15	4.70 - 8.08	4.46	3.39 - 5.91	3.44	2.54 - 4.71
Vasopressor	0.81	0.64 -1.02	0.91	0.73 - 1.12	1.04	0.84 - 1.30
RRT	1.11	0.65 - 1.88	1.20	0.70 - 2.09	1.27	0.70 - 2.43
CCI	1.02	0.95 - 1.09	1.05	0.98 - 1.12	1.02	0.96 - 1.09
Age	1.00	0.99 - 1.01	0.99	0.99 - 1.00	1.00	0.99 - 1.00

OR - odds ratio; 95%CI - 95% confidence interval; ECOG - Eastern Cooperative Oncology Group; PS - performance status; SOFA - Sequential Organ Failure Assessment; MV - mechanical ventilation; RRT - renal replacement therapy; CCI - Charlson Comorbidity Index.

Table 3S - Estimated odds ratios and 95% confidence intervals of individual predictors for all locoregional solid tumors

Variable	28-day		90-day		360-day	
	OR	CI95%	OR	CI95%	OR	CI95%
ECOG-OS						
0 - 1	Ref		Ref		Ref	
2	3.41	2.16 - 5.37	3.79	2.58 - 5.57	3.16	2.22 - 4.52
3 - 4	5.93	3.74 - 9.44	6.25	4.13 - 9.50	4.78	3.19 - 7.24
SOFA	1.11	1.02 - 1.20	1.08	1.00 - 1.16	1.12	1.04 - 1.19
MV	4.39	2.70 - 7.15	3.34	2.11 - 5.30	2.94	1.87 - 4.65
Vasopressor	1.86	1.20 - 2.87	1.70	1.16 - 2.49	1.53	1.08 - 2.16
RRT	0.55	0.13 - 1.90	0.77	0.23 - 2.44	1.33	0.42 - 4.72
CCI	1.17	1.04 - 1.30	1.14	1.03 - 1.26	1.10	1.00 - 1.21
Age	1.00	0.99 - 1.01	1.00	0.98 - 1.02	1.00	0.99 - 1.02

OR - odds ratio; 95%CI - 95% confidence interval; ECOG - Eastern Cooperative Oncology Group; PS - performance status; SOFA - Sequential Organ Failure Assessment; MV - mechanical ventilation; RRT - renal replacement therapy; CCI - Charlson Comorbidity Index.

Table 4S - Estimated odds ratios and 95% confidence intervals of individual predictors for metastatic solid tumors

	28-day		90-day		360-day	
	**OR**	**CI95%**	**OR**	**CI95%**	**OR**	**CI95%**
ECOG-OS						
0 - 1	Ref		Ref		Ref	
2	3.41	2.16 - 5.37	2.75	2.15 - 3.53	2.37	1.79 - 3.17
3 - 4	5.93	3.74 - 9.44	3.57	2.65 - 4.84	3.24	2.27 - 4.73
SOFA	1.11	1.02 - 1.20	1.21	1.15 - 1.27	1.20	1.13 - 1.27
MV	4.39	2.70 - 7.15	4.63	3.12 - 7.01	3.10	1.93 - 5.16
Vasopressor	1.86	1.20 - 2.87	0.62	0.47 - 0.83	0.86	0.63 - 1.19
RRT	0.55	0.13 - 1.90	0.48	0.22 - 1.07	0.55	0.23 - 1.47
CCI	1.17	1.04 - 1.30	1.05	0.96 - 1.16	1.03	0.93 - 1.15
Age	1.00	0.98 - 1.02	0.99	0.98 -1.00	0.99	0.98 - 1.00

OR - odds ratio; 95%CI - 95% confidence interval; ECOG - Eastern Cooperative Oncology Group; PS - performance status; SOFA - Sequential Organ Failure Assessment; MV - mechanical ventilation; RRT - renal replacement therapy; CCI - Charlson Comorbidity Index.

Table 5S - Estimated odds ratios and 95% confidence intervals of individual predictors for hematological malignancy

	28-day		90-day		360-day	
	**OR**	**CI95%**	**OR**	**CI95%**	**OR**	**CI95%**
ECOG-PS						
0 - 1	Ref		Ref		Ref	
2	3.08	1.36 - 6.88	2.15	1.03 - 4.44	2.82	1.43 - 5.71
3 - 4	7.68	3.36 - 17.75	9.89	4.49 - 23.09	6.49	2.91 - 15.84
SOFA	1.18	1.06 - 1.32	1.26	1.14 - 1.40	1.21	1.10 - 1.33
MV	17.94	8.31 - 41.20	8.23	3.89 - 18.33	7.56	3.41 - 18.15
Vasopressor	1.04	0.51 - 2.07	1.02	0.55 - 1.84	0.74	0.42 - 1.29
RRT	1.59	0.57 - 4.55	4.44	1.43 - 17.00	2.25	0.72 - 8.58
CCI	0.86	0.68 - 1.06	0.87	0.71 - 1.04	0.85	0.71 - 1.00
Age	1.02	0.99 - 1.05	1.01	0.99 - 1.03	1.01	0.99 - 1.03

OR - odds ratio; 95%CI - 95% confidence interval; ECOG - Eastern Cooperative Oncology Group; PS - performance status; SOFA - Sequential Organ Failure Assessment; MV - mechanical ventilation; RRT - renal replacement therapy; CCI - Charlson Comorbidity Index.
