# Supplementary material for: Differences in the relative importance of predictors of short- and long-term mortality among critically ill patients with cancer
Source: Crit Care Sci. 2024 Nov 11;36:e20240149en. doi: 10.62675/2965-2774.20240149-en (PMC11634285; doi:10.62675/2965-2774.20240149-en)
Supplement: Translation

# Translation: Diferenças na importância relativa dos preditores de mortalidade em curto e longo prazo de pacientes críticos com câncer

**DOI:** 10.62675/2965-2774.20240149-pt

**Authors:** Carla Marchini Dias da Silva, Bárbara Beltrame Bettim, Bruno Adler Maccagnan Pinheiro Besen, Antônio Paulo Nassar

**Affiliations:** 1 São Paulo SP Brasil Unidade de Terapia Intensiva, A.C. Camargo Cancer Center - São Paulo (SP), Brasil.; 2 São Paulo SP Brasil Centro Internacional de Pesquisa, A. C. Camargo Cancer Center - São Paulo (SP), Brasil.

**Keywords:** Estado terminal, Neoplasias, Resultados de cuidados críticos, Mortalidade, Prognóstico, Unidades de terapia intensiva

## Abstract

**Objetivo:**

Identificar a importância relativa de variáveis clínicas presentes na admissão na unidade de terapia intensiva na mortalidade em curto e longo prazo de pacientes críticos com câncer após admissão não planejada.

**Métodos:**

Trata-se de estudo de coorte retrospectivo de pacientes com câncer com admissão não planejada na unidade de terapia intensiva de janeiro de 2017 a dezembro de 2018. Desenvolvemos modelos para analisar a importância relativa de preditores conhecidos de mortalidade em pacientes com câncer admitidos na unidade de terapia intensiva na mortalidade em 28, 90 e 360 dias, tanto em toda a coorte quanto na estratificada pelo tipo de câncer.

**Resultados:**

Dos 3.592 pacientes, 3.136 (87,3%) tinham tumores sólidos, com doença metastática observada em 60,8% deles. Foi registrada a morte de 1.196 (33,3%), 1.738 (48,4%) e 2.435 pacientes (67,8%) em 28, 90 e 360 dias, respectivamente. A maior contribuição na mortalidade de todos os pacientes foi o comprometimento da capacidade funcional em curto prazo e, em curto e longo prazo, nos subgrupos de pacientes com tumores sólidos. O SOFA na admissão foi importante para prever a mortalidade apenas de pacientes com tumores sólidos metastáticos e neoplasias hematológicas. O uso de vasopressores e terapia de substituição renal teve uma pequena importância na predição de mortalidade em todos os momentos analisados após considerar o SOFA.

**Conclusão:**

Os profissionais de saúde devem considerar a capacidade funcional, o uso de ventilação mecânica e a gravidade da doença ao discutirem o prognóstico, as preferências de cuidados e o planejamento de cuidados no fim da vida com os pacientes ou seus familiares durante a internação na unidade de terapia intensiva.

## INTRODUÇÃO

A taxa de mortalidade por câncer vem diminuindo em todo o mundo, com redução geral de 33% desde 1991.^(1)^ Esse progresso reflete cada vez mais as melhorias na detecção precoce, assim como os avanços nos protocolos de tratamento, incluindo o desenvolvimento de terapias-alvo.^(2)^ No entanto, essas mudanças também foram acompanhadas por um número maior de pacientes suscetíveis a complicações ameaçadoras à vida. Consequentemente, um número crescente de pacientes com câncer está em risco de ser internado na unidade de terapia intensiva (UTI).^(3,4)^ O risco de internação na UTI devido a complicações clínicas do tratamento do câncer é de 15,0%,^(5)^ e até 22,5% dos pacientes com neoplasias hematológicas precisam de cuidados na UTI durante o primeiro ano após o diagnóstico.^(6)^

As mudanças recentes no cenário da oncologia têm modificado as indicações atuais de internação na UTI e o prognóstico associado a pacientes críticos com câncer. Embora os desfechos de pacientes críticos com câncer tenham melhorado na última década, os cuidados na UTI estão associados à morbidade persistente após a alta,^(7)^ e os pacientes com câncer admitidos em UTI considerados por intensivistas como internações potencialmente inapropriadas têm mortalidade de 97,6% em 1 ano.^(8)^

A decisão de internar ou não um paciente na UTI pode ser um difícil desafio clínico e ético. Vários estudos avaliaram a influência de fatores de risco associados a desfechos em curto prazo (mortalidade na UTI, no hospital e em 30 dias),^(9,10)^ mas poucos avaliaram seu efeito na mortalidade em longo prazo em pacientes com câncer após a admissão na UTI. Uma melhor compreensão dos fatores capazes de influenciar os desfechos desses doentes pode ajudar os profissionais de saúde a melhorar o processo de tomada de decisão compartilhada. Para ajudar a preencher essas lacunas, nosso estudo objetivou identificar a importância relativa de variáveis clínicas presentes na admissão na UTI na mortalidade em curto e longo prazo de pacientes críticos com câncer após uma admissão não planejada.

## MÉTODOS

### Desenho e contexto do estudo

Trata-se de uma análise secundária de um estudo de coorte retrospectivo com dados coletados prospectivamente, realizado de janeiro de 2017 a dezembro de 2018 em um centro dedicado ao câncer no Brasil.^(11)^ O Comitê de Ética em Pesquisa Científica do *A. C. Camargo Cancer Center* aprovou o estudo e dispensou a necessidade de Termo de Consentimento Livre e Esclarecido, uma vez que todos os dados foram totalmente anonimizados antes de os pesquisadores obterem acesso (CAAE: 05694819.3.0000.5432).

Nosso centro é um hospital terciário em São Paulo, com 447 leitos para pacientes com câncer, e atende a aproximadamente 1.850 pacientes clínicos e cirúrgicos por mês. O Departamento de Terapia Intensiva consiste em cinco UTIs gerais e tem 50 leitos. Todas as UTIs têm o mesmo perfil e podem admitir pacientes do departamento de emergência, do centro cirúrgico, das enfermarias ou transferidos de outro hospital. Não há política de triagem de UTI no hospital. Depois que o paciente é admitido, a equipe da UTI é a principal responsável pela assistência ao doente. As decisões relativas à instituição de suporte avançado de vida e da alta da UTI são tomadas pelo intensivista responsável. As decisões acerca da suspensão ou retirada de suporte avançado de vida são tomadas após deliberações entre intensivistas, oncologistas/hematologistas e pacientes ou seus responsáveis legais.

Relatamos este estudo de acordo com a declaração *Transparent Reporting of a Multivariable Prediction Model for Individual Prognosis or Diagnosis* (TRIPOD).^(12)^

### População do estudo e coleta de dados

Nessa análise, incluímos todos os pacientes adultos (idade ≥ 18 anos) com internações não planejadas na UTI. Excluímos pacientes sem câncer e aqueles readmitidos na UTI durante a mesma internação hospitalar.

Obtivemos os dados dos pacientes em bancos de dados locais e prontuários médicos eletrônicos. Foram coletados os seguintes dados demográficos e clínicos: tipo de câncer (sólido locorregional, sólido metastático ou hematológico); local do câncer sólido ou tipo de neoplasia hematológica; capacidade funcional do *Eastern Cooperative Oncology Group* (ECOG-PS)^(13)^ antes da admissão hospitalar (registrado na última consulta ambulatorial); Índice de Comorbidade de Charlson (ICC),^(14)^ *Sequential Organ Failure Assessment* (SOFA)^(15)^ e *Simplified Acute Physiology Score 3* (SAPS 3)^(16)^ na admissão; e motivo da internação na UTI.

Além disso, coletamos dados do uso de suporte de órgãos (drogas vasoativas [DVA], ventilação mecânica [VM] invasiva, terapia de substituição renal [TSR]), duração da internação na UTI e no hospital e mortalidade em 28, 90 e 360 dias.

### Preditores e desfechos

Incluímos preditores bem conhecidos de mortalidade em pacientes com câncer internados na UTI: tipo de câncer (sólido locorregional, sólido metastático ou hematológico), idade, carga de comorbidade avaliada pelo ICC (sem pontos relacionados ao câncer), SOFA, necessidade de DVA, VM e TSR e ECOG-PS.^(3,17–19)^

A importância relativa dos preditores foi avaliada em relação à mortalidade em 28, 90 e 360 dias após a admissão na UTI, tanto em toda a amostra quanto estratificada pelo tipo de câncer.

### Análise estatística

#### Desenvolvimento dos modelos

As previsões dos modelos de mortalidade foram desenvolvidas na coorte da população estudada. A incidência de mortalidade foi regredida em função da idade e das variáveis preditoras predefinidas. Todas as variáveis preditoras numéricas (idade, ICC e SOFA) foram modeladas como variáveis continuas.^(20)^ Avaliamos a importância relativa da variável com o teste do qui quadrado de Wald de cada variável em relação ao teste do qui quadrado total no modelo multivariável. Os coeficientes de regressão do modelo (e as razões de chances) foram derivados, mas como não eram a apresentação primária de interesse, não realizamos procedimentos de encolhimento ou seleção de variáveis. A mesma estratégia de modelagem foi aplicada nas quatro coortes (coorte completa, cânceres locorregionais sólidos, cânceres metastáticos sólidos e cânceres hematológicos) e em cada momento de mortalidade (28, 90 e 360 dias). Avaliamos a validação aparente desses modelos com a estatística C (área sob a curva Características de Operação do Receptor)^(21)^ e gráficos de calibração.^(22)^ Também realizamos a validação interna com estimativas de discriminação corrigidas para otimismo após 200 reamostragens por *bootstrap*.^(20)^

Das variáveis selecionadas usadas nos modelos, faltaram apenas os dados do ECOG (11%). Assumimos que os dados estavam ausentes de forma aleatória e aplicamos uma técnica de imputação múltipla usando equações encadeadas (MICEs).^(23)^

Todas as análises foram realizadas usando o *Statistical Package for the Social Sciences* (SPSS) versão 20) e o R versão 4.3.0 (R Foundation for Statistical Computing).

## RESULTADOS

### Características da população do estudo

De janeiro de 2017 a dezembro de 2018, 8.064 pacientes foram admitidos nas UTIs. Desses, 3.592 pacientes eram aptos para as análises (Figura 1S - Material suplementar). A idade média foi de 65 (± 15) anos, 1.747 (48,6%) eram mulheres e 3.136 (87,3%) tinham tumores sólidos. Os tumores sólidos colorretal (14,8%), pulmonar (12,1%), mama (11,1%) e de cabeça e pescoço (7,8%) foram os mais comuns. Observou-se doença metastática em 60,8% desses pacientes. O linfoma não Hodgkin (33,9%), o mieloma múltiplo (18%) e a leucemia aguda (17,7%) foram as neoplasias hematológicas mais comuns.

Registrou-se a admissão de 3.205 pacientes (89,2%) devido a complicações clínicas e 387 (10,8%) após cirurgias de urgência. Os principais motivos das internações clínicas foram sepse e choque séptico (30,5%), insuficiência respiratória aguda (IRpA) (19,1%) e distúrbios neurológicos (6,5%). A média do SOFA foi de 3,0 (± 3) pontos, a mediana do ICC foi de 7,0 (5 - 8) e a média do SAPS 3 foi de 64 (± 15).

A mediana da duração da internação na UTI e no hospital foi de 5 (2 - 10) e 12 (6 - 24) dias, respectivamente. Ao todo, 1.196 (33,3%), 1.738 (48,4%) e 2.435 pacientes (67,8%) morreram em 28, 90 e 360 dias, respectivamente (Tabela 1).

**Tabela 1:** Características dos pacientes com internação não planejada na unidade de terapia intensiva

Variáveis		
Sexo feminino		1.747 (48,6)
Idade (anos)		62 ± 15
ICC		7 [5 - 8]
ECOG*		3.297 (97,6)
	0	727 (22,1)
	1	1.365 (41,4)
	2	694 (21,0)
	3	440 (13,3)
	4	71 (2,2)
SOFA		3 ± 3
SAPS 3		64 ± 15
Tumor sólido		3.136 (87,3)
Tumor sólido, metastático		1.909 (60,8)
Neoplasia hematológica		456 (12,7)
Tipo de admissão		
	Clínica	3.205 (89,2)
	Cirurgia de urgência	387 (10,8)
Motivo da admissão		
	Sepse e choque séptico	979 (30,54)
	Insuficiência respiratória aguda	613 (19,13)
	Estado mental alterado	209 (6,52)
	Arritmia	147 (4,59)
Terapia de substituição renal		92 (2,6)
Ventilação mecânica		533 (14,8)
Drogas vasoativas		1.082 (30,1)
Duração da internação na UTI (dias)		5 [2 - 10]
Duração da internação hospitalar (dias)		10 [5 - 19]
Mortalidade em 28 dias		1.196 (33,3)
Mortalidade em 90 dias		1.738 (48,4)
Mortalidade em 360 dias		2.435 (67,8)

ICC - Índice de Comorbidade de Charlson; ECOG - *Eastern Cooperative Oncology Group*; SOFA - *Sequential Organ Failure Assessment*; SAPS - *Simplified Acute Physiological Score*; UTI - unidade de terapia intensiva.

* Faltaram dados de 395 (11%) pacientes. Resultados expressos como n (%), média ± desvio-padrão ou mediana [intervalo interquartil].

### Importância relativa de diferentes preditores na mortalidade de pacientes internados em uma unidade de terapia intensiva

#### Todos os pacientes

A capacidade funcional foi a variável mais importante associada à mortalidade em 90 dias em todos os pacientes e teve a maior importância relativa, assim como a VM em 28 dias. Sua importância relativa aumentou de 19,8% em 28 dias, para 34,5% em 90 dias, e, posteriormente, diminuiu para 33,8% em 360 dias. Por outro lado, o tipo de câncer foi a variável mais importante associada à mortalidade em 360 dias. A importância relativa do câncer aumentou de 16,6% em 28 dias para 29,4% em 90 dias, e 40,5% em 360 dias. A importância relativa da VM diminuiu de 19,8% em 28 dias para 16,5% e 10% em 90 e 360 dias, respectivamente. A importância relativa do SOFA na admissão na predição de mortalidade em 28 dias foi de 9,1%, que, posteriormente, aumentou para 11,8% em 90 dias, e, depois, diminuiu para 10,3% em 360 dias. O uso de DVA, TSR, idade e outras comorbidades além do câncer tiveram pouca importância relativa na predição de mortalidade (Figura 1A).

**Figura 1:** Importância relativa do preditor em cada momento na mortalidade estratificada por tipo de câncer.

A importância relativa de cada preditor é estimada como a razão entre o teste do qui quadrado de Wald para o preditor em relação ao modelo geral do teste qui quadrado.

ECOG-PS - capacidade funcional do *Eastern Cooperative Oncology Group*; VM - ventilação mecânica; SOFA - *Sequential Organ Failure Assessment*; TSR - terapia de substituição renal; ICC - Índice de Comorbidade de Charlson.

#### Pacientes com tumores sólidos locorregionais

A capacidade funcional foi a variável mais importante associada à mortalidade em 28, 90 e 360 dias em pacientes com tumores sólidos locais/regionais. Sua importância relativa aumentou de 13,7% em 28 dias para 26,6% e 42,8% em 90 dias e 360 dias, respectivamente. A VM foi a segunda variável mais importante na predição de mortalidade, com valores de 9,6%, 10,2% e 12,8%, em 28, 90 e 360 dias, respectivamente. A importância relativa do SOFA na admissão na predição de mortalidade em 28 e 90 dias foi de 1,8%, aumentando para 4,6% em 360 dias. O uso de vasopressores teve importância relativa de 2,3%, 2,3% e 1,8% na predição de mortalidade, em 28, 90 e 360 dias, respectivamente. As comorbidades, que não o câncer, tiveram importância relativa de 1,5% na predição de mortalidade em 28 dias, com importância insignificante em 90 e 360 dias.

A TSR e a idade tiveram pouca importância relativa na predição de mortalidade durante todos os períodos do estudo (Figura 1B).

#### Pacientes com tumores sólidos metastáticos

A capacidade funcional foi a variável mais importante associada à mortalidade em 28, 90 e 360 dias em pacientes com tumores sólidos metastáticos. Sua importância relativa aumentou de 33,1% em 28 dias para 50,1% em 90 dias e, posteriormente, diminuiu para 27,1% em 360 dias. A VM foi a segunda variável mais importante para predizer a mortalidade em 28 dias, com redução na importância de 26,3% para 18,3% e para 4,3% em 28, 90 e 360 dias, respectivamente. O SOFA foi a segunda variável mais importante na predição de mortalidade em 90 e 360 dias. Sua importância relativa aumentou de 21,6% em 28 dias, para 23,4% em 90 dias e, posteriormente, diminuiu para 9,5% em 360 dias. O uso de vasopressores teve importância relativa de 4,4% e 3,5% em 28 e 90 dias, respectivamente. A TSR, a idade e as comorbidades que não o câncer tiveram importância relativa inferior a 1,5% na predição de mortalidade durante todos os períodos do estudo (Figura 1C).

#### Pacientes com neoplasias hematológicas

A VM foi a variável mais importante associada à mortalidade em todos os períodos do estudo. Sua importância relativa diminuiu de 31,9% em 28 dias, para 22,5% em 90 dias, e, posteriormente, aumentou para 26,5% em 360 dias. A capacidade funcional foi a segunda variável mais importante associada à mortalidade, e sua importância relativa aumentou de 10,1% em 28 dias, para 20,8% e 24,5% em 90 e 360 dias, respectivamente. A importância relativa do SOFA na admissão para predizer a mortalidade aumentou de 6,1% em 28 dias, para 15,5% e 22,3% em 90 dias e 360 dias, respectivamente. Outras comorbidades além do câncer tiveram importância relativa de 1,5%, 2,6% e 7,7% para predizer a mortalidade em 28, 90 e 360 dias, respectivamente. A importância relativa da TSR aumentou de 0,9% em 28 dias, para 5,6% em 90 dias, e, depois, diminuiu para 3,6% em 360 dias. O uso de DVA e a idade tiveram importância relativa inferior a 1% para prever a mortalidade em todos os períodos do estudo (Figura 1D).

Todos os modelos apresentaram boa discriminação (Tabela 1S - Material Suplementar). As razões de chances estimadas e os intervalos de confiança de 95% são apresentados no arquivo suplementar (Tabelas 2S a 5S - Material Suplementar).

## DISCUSSÃO

Os resultados deste estudo mostram que o comprometimento da capacidade funcional foi a maior contribuição para a mortalidade em curto prazo de todos os pacientes, e em curto e longo prazo para os subgrupos de doentes com tumores sólidos. Em pacientes com neoplasias hematológicas, o uso de VM foi a variável mais importante associada à mortalidade em todos os períodos do estudo. O SOFA na admissão foi relativamente importante na previsão de mortalidade apenas para pacientes com tumores sólidos metastáticos e neoplasias hematológicas. O uso de DVA e da TSR teve pequena importância relativa na predição de mortalidade em todos os momentos analisados após considerar o SOFA.

Nossa análise confirmou o impacto da capacidade funcional comprometida em desfechos desfavoráveis em pacientes com câncer, o que contribui para o maior risco de mortalidade, especialmente em pacientes com tumores sólidos. A funcionalidade comprometida preexistente está associada a desfechos mais desfavoráveis durante e após a internação na UTI.^(3,7,19)^ Estudos recentes indicaram que a recuperação de uma doença crítica deteriora a capacidade funcional basal em pacientes com câncer,^(7)^ o que pode afetar a elegibilidade a tratamentos em andamento^(24,25)^ e estar associado à menor sobrevida até um ano após a alta hospitalar.^(24,26)^

Embora estudos recentes tenham demonstrado que as taxas de mortalidade em pacientes com câncer que fizeram uso de VM durante a internação na UTI melhoraram ao longo do tempo,^(3,10)^ nossos achados indicam que a VM ainda é um fator prognóstico importante. De fato, em pacientes com neoplasias hematológicas, o uso de VM representou a variável associada à maior importância relativa na mortalidade em todos os pontos analisados. A IRpA ainda é a principal causa de internação na UTI desses pacientes,^(27)^ e a necessidade de VM continua a ser um determinante de desfechos mais desfavoráveis,^(27,28)^ apesar das melhorias no prognóstico de pacientes críticos com neoplasias hematológicas ao longo do tempo.^(3,10,29)^ A alta mortalidade levou os médicos a considerarem a ventilação não invasiva (VNI) para melhorar o prognóstico dessa população. No entanto, a melhor estratégia inicial de manejo ventilatório desses pacientes ainda suscita dúvidas e incertezas.^(26,29,30,31)^ Embora estudos anteriores tenham demonstrado que a VNI possa beneficiar pacientes com neoplasias hematológicas com IRpA,^(30,31)^ ela pode falhar em aproximadamente 50% dos pacientes,^(28,30)^ e aqueles com falha na VNI têm prognóstico semelhante ou até mais grave do que pacientes que receberam VM inicialmente.^(28,30,31)^ A admissão precoce na UTI, a investigação etiológica da insuficiência respiratória hipoxêmica e a identificação dos candidatos ideais à VNI de pacientes onco-hematológicos podem ajudar a maximizar a eficácia dessa ferramenta em casos de disfunção respiratória nessa população.^(25,27)^

Nosso estudo sugere que a idade e a carga de comorbidades além daquelas associadas ao câncer tiveram efeitos insignificantes no risco de mortalidade em todos os momentos analisados. Esses achados não são inesperados. Em comparação com coortes indiferenciadas de pacientes com câncer admitidos na UTI, pacientes muito idosos com câncer admitidos na UTI têm taxas de mortalidade semelhantes em curto e longo prazo.^(32,33)^ Embora a carga de comorbidades seja um preditor significativo de mortalidade em longo prazo em pacientes em estado crítico,^(34)^ grande parte do efeito da comorbidade neste estudo foi devido ao câncer em si.^(32)^ Curiosamente, a carga de comorbidades teve alguma importância relativa nos desfechos em 360 dias dos pacientes com câncer hematológico em nossa coorte.

O SOFA na admissão foi relativamente importante para a mortalidade apenas em pacientes com tumores sólidos metastáticos e neoplasias hematológicas, e o subgrupo de pacientes em que a VM foi usada teve um peso proporcional relativamente alto na previsão de mortalidade, embora a importância relativa do SOFA tenha aumentado em longo prazo nas neoplasias hematológicas, mas diminuído nos tumores sólidos metastáticos. Na população geral, essa associação entre o SOFA e os desfechos mais desfavoráveis é mais forte em curto prazo e diminui com o passar do tempo, enquanto a importância relativa da carga de comorbidades aumenta em longo prazo.^(35)^ Em nosso estudo, embora a carga de comorbidades não oncológica não tenha sido associada à sobrevida em longo prazo, as características do câncer e a capacidade funcional desfavorável estavam associadas.

Estudos demonstraram que o uso de vasopressores e a necessidade de TSR na internação na UTI estão independentemente associados ao aumento da mortalidade, e os pacientes com câncer que necessitam de diálise têm taxas de mortalidade intra-hospitalar entre 51 e 90%.^(36–38)^ Curiosamente, o uso de vasopressores e TSR contribuiu relativamente pouco para a mortalidade, mesmo a mortalidade em 28 dias. Isso provavelmente ocorreu porque nosso modelo levou em conta o SOFA. Nossos resultados sugerem que os desfechos são afetados não apenas por fatores isolados, mas por uma interação complexa entre os diferentes tipos de câncer, o nível de suporte de órgãos, a gravidade da doença aguda e a capacidade funcional. Essa observação foi feita em outras populações específicas, como pacientes muito idosos com pneumonia submetidos à VM invasiva ou VNI^(39)^ ou pacientes graves com trauma, em que o SOFA é um preditor composto altamente confiável de desfecho.^(40)^ Portanto, a necessidade de DVA ou TSR não devem ser fatores isolados para não escalonar o nível de cuidados ou para iniciar cuidados de conforto e deliberar sobre cuidados de fim de vida na UTI, especialmente em pacientes com câncer e com boa capacidade funcional.

O nosso estudo tem alguns pontos fortes. Primeiro, avaliamos especificamente pacientes com câncer, nos quais os médicos podem ter dúvidas quanto à adequação da internação na UTI. Pesquisas de alta qualidade acerca da mortalidade em curto e longo prazo de pacientes com câncer com internação não planejada na UTI são essenciais para controlar as expectativas de desfecho dos profissionais de saúde, pacientes e seus familiares. Em segundo lugar, analisamos a mortalidade e os fatores prognósticos agrupando os pacientes conforme os diferentes tipos de câncer (sólido locorregional, sólido metastático ou hematológico). Nossos achados de diferenças na importância dos fatores prognósticos nos diferentes tipos de câncer indicam que o prognóstico de um paciente deve ser considerados sem a fusão desses grupos sob a alcunha de câncer isoladamente. Além disso, a capacidade dos fatores prognósticos combinados de prever os efeitos de diversas variáveis clínicas presentes na admissão na UTI quanto à mortalidade em curto e longo prazo de pacientes críticos com câncer é notável e merece ser validada em estudos futuros.

Nosso estudo também tem várias limitações. Primeiro, por ser um estudo de centro único realizado em um centro de câncer exclusivo, seus resultados podem não ser generalizáveis. Em segundo lugar, não foi possível incluir dados relativos à fragilidade, que está independentemente associada aos desfechos e ao uso de recursos em pacientes em estado crítico e poderia ter influenciado nos resultados.^(41)^ Em terceiro lugar, embora tenhamos excluído pacientes com diretivas antecipadas definidas quanto à não instituição de medidas invasivas antes da admissão na UTI, não consideramos as decisões realizadas durante a internação na UTI, o que poderia ter impactado a mortalidade em curto e, possivelmente, em longo prazo.^(42)^ Em quarto lugar, não coletamos dados do intervalo entre a admissão na UTI, o desenvolvimento de disfunções orgânicas e o início de terapias de suporte orgânico. Entretanto, em nosso hospital, os leitos de UTI estão prontamente à disposição dos pacientes que precisam de suporte orgânico. Em quinto lugar, não coletamos dados relativos à condição socioeconômica, que está associada a desfechos e poderia ter repercutido nos resultados. A condição socioeconômica mais baixa, tanto em nível individual quanto em nível demográfico, está associada a um tratamento de câncer abaixo do ideal e à redução da sobrevida,^(43)^ uma vez que os pacientes com câncer de áreas socioeconomicamente desfavorecidas têm acesso limitado a serviços de triagem e tratamento e tendem a ter uma doença mais avançada na apresentação.^(44)^ Em sexto lugar, não coletamos dados de qualidade de vida. A carga significativa de deficiências físicas, cognitivas e de saúde mental após a alta da UTI, com o aumento da vulnerabilidade a desfechos adversos, como mortalidade e reinternação, não pode ser ignorada.^(45)^ Por fim, não levamos em conta outras características individuais do câncer que podem ser usadas na tomada de decisões individuais, que provavelmente devem ser consideradas além dos nossos resultados observados.

## CONCLUSÃO

Nosso estudo revelou que as variáveis clínicas presentes na admissão na unidade de terapia intensiva apresentam interação complexa e têm importância diferente na mortalidade de pacientes com câncer após a admissão não planejada na unidade de terapia intensiva, de acordo com diferentes tipos de câncer e diferentes marcos de mortalidade. Os profissionais de saúde devem considerar a capacidade funcional, o uso de ventilação mecânica e a gravidade da doença ao abordarem o prognóstico, as preferências de cuidados e o planejamento de cuidados de fim de vida com os pacientes ou seus familiares durante a internação na unidade de terapia intensiva. No entanto, ainda são necessários mais estudos para caracterizar os preditores de outros desfechos em longo prazo em pacientes com câncer em estado crítico.

## Data Availability

Os conjuntos de dados usados e analisados em nosso estudo estão acessíveis mediante solicitação razoável ao autor correspondente.
